# Trained Scent Dog Detection and GC-MS Analysis of Volatile Organic Compounds from Murine Coronavirus-Infected Cell Cultures

**DOI:** 10.3390/ani16040647

**Published:** 2026-02-18

**Authors:** Agata Kokocińska-Alexandre, Martyna Woszczyło, Michał Dzięcioł, Agata Kublicka, Adam Szumowski, Jacek Łyczko, Katarzyna Barłowska, Antoni Szumny, Marcin J. Skwark, Anna Karolina Matczuk

**Affiliations:** 1 Institute of Biological Bases of Animal Production, Faculty of Animal Sciences and Bioeconomy, University of Life Sciences in Lublin, 20-950 Lublin, Poland; akokocinska@ethoplanet.com; 2 Department of Reproduction and Clinic for Farm Animals, The Faculty of Veterinary Medicine, Wrocław University of Environmental and Life Sciences, 50-366 Wrocław, Poland; michal.dzieciol@upwr.edu.pl; 3 VetAI.bio Ltd., London UB9 5NJ, UK; martyna@vetai.bio; 4 Department of Pathology, Division of Microbiology, The Faculty of Veterinary Medicine, Wrocław University of Environmental and Life Sciences, 50-375 Wrocław, Poland; agata.kublicka@upwr.edu.pl; 5 Department of Food Chemistry and Biocatalysis, The Faculty of Biotechnology, Wrocław University of Environmental and Life Sciences, 50-375 Wrocław, Poland; 115025@student.upwr.edu.pl (A.S.); jacek.lyczko@upwr.edu.pl (J.Ł.); antoni.szumny@upwr.edu.pl (A.S.); 6 Institute of Genetics and Animal Biotechnology, Polish Academy of Sciences, 05-552 Jastrzębiec, Poland; k.barlowska@igbzpan.pl; 7 InstaDeep Ltd., London W2 1AS, UK

**Keywords:** viral infection detection, scent detection dogs, volatile organic compounds, VOC, murine hepatitis virus, coronavirus, GC-MS, canine olfaction, machine learning classifier

## Abstract

Volatile organic compounds (VOCs) are increasingly recognized as metabolic byproducts of viral infection and may serve as olfactory cues detectable by trained scent dogs. This study examined whether dogs could distinguish cell culture samples infected with murine hepatitis virus strain 1 (MHV-1), a biosafety level 2 coronavirus model, from uninfected controls. Parallel chemical analysis using gas chromatography–mass spectrometry (GC-MS) identified 14 VOCs in infected and 12 in control samples. Notably, 3-heptanone and 1-nonanol were unique to infected samples, while others such as acetophenone, nonanal, decanal, and benzaldehyde were significantly elevated—often by 1.5 to 3 times—in infected cultures. Two trained dogs demonstrated high detection sensitivity (0.95) for infected samples compared to a previously trained odor cinnamon group (0.88) and responded with shorter latency (*p* = 0.04), suggesting perceptual salience of infection-related VOCs. Reliable detection required pooled volumes (~600 µL), suggesting a threshold effect related to VOC concentration. Additionally, a Random Forest-based machine learning classifier trained on the GC-MS-obtained VOC profiles achieved a cross-validated accuracy of 0.82 (SD = 0.25). These findings suggest that dogs use quantitative VOC differences, rather than unique compounds, for detection. The study provides a validated experimental framework for olfactory diagnostics of viral infections and highlights the potential of scent dogs as non-invasive biosensors in both veterinary and public health contexts.

## Simple Summary

Detecting viral infections quickly and accurately is critical for protecting public and animal health. In this study, we explored whether specially trained dogs can detect signs of viral infection by scent alone. We used a harmless murine virus that infects cells in the lab. The dogs were trained to tell the difference between virus-infected and uninfected samples. At the same time, we used a chemical method to identify which specific scent molecules were present. Some of these molecules were only found in infected samples, while others were present at much higher concentrations than in uninfected samples. The dogs were able to correctly identify virus-infected samples in most cases. A computational method using the chemical data also correctly identified infected samples with high accuracy. This work supports the idea that trained dogs can help detect infections through scent and may improve future non-invasive methods for viral detection.

## 1. Introduction

Volatile organic compounds (VOCs) are chemicals characterized by their high vapor pressure at ambient temperature. These substances are recognized as odorants by both humans and animals because they bind to olfactory epithelium receptors, located in the distal part of the nasal cavity.

Endogenously produced VOCs result from the metabolic processes within the body, and variations in these compounds can indicate aging and signal specific diseases [1,2,3]. Additionally, the microbiome of both humans and animals generates distinct VOC profiles that may shift under various conditions [4,5].

Prior to the SARS-CoV-2 pandemic, there was limited research on VOC production in the context of viral infections, either in cell cultures or animal models. Since virions (virus particles) lack inherent metabolic activity—and their components (lipids, nucleic acids, and proteins) are generally considered odorless—the generation of VOCs associated with viral infection is likely linked to metabolic alterations in infected host cells [6]. These changes may be due to such processes as viral replication, oxidative stress, and the initiation of apoptosis. Upon virus infection the cellular metabolism changes, as metabolomic studies indicate that most of the viruses induce glycolysis, changes in fatty acid synthesis and/or glutaminolysis. These changes are required for induction of specific host metabolic pathways necessary for replication and rapid synthesis of viral components, such as nucleic acids and proteins, for successful virus assembly and consequent spread [7].

Studies on cultured cells infected with human respiratory viruses, including respiratory syncytial virus (RSV) and influenza A virus (IAV), demonstrated that viral infection induces reproducible changes in the profile of VOCs emitted from the cell cultures [8].

These changes are not characterized by the appearance of unique virus-specific compounds, but rather by shifts in the relative abundances and proportions of common volatile metabolites, such as alkanes, aldehydes, ketones, and alcohols. In particular, hydrocarbons were found to be more abundant in infected cultures, a pattern likely linked to oxidative stress and lipid peroxidation induced by viral replication. Machine learning analyses were able to discriminate between infected and uninfected samples based on the relative intensities of these volatiles with AUROCs (areas under receiver-operator curves) ranging from 0.78 to 0.84, underscoring the diagnostic potential of the collective VOC fingerprint rather than reliance on individual marker compounds [8].

Studies analyzing exhaled breath have identified distinct VOC profiles associated with SARS-CoV-2 infection [9,10]. In adults, gas chromatography–ion mobility spectrometry (GC-IMS) revealed that patients with COVID-19 exhibited elevated concentrations of aldehydes (ethanal, octanal, and heptanal), ketones (acetone and 2-butanone), and methanol, distinguishing them from patients with other respiratory or cardiac conditions with high accuracy (AUROC 0.87–0.91) [10]. Similarly, pediatric studies using two-dimensional gas chromatography and time-of-flight mass spectrometry (GCxGC-ToF-MS) confirmed that three medium-chain aldehydes, octanal, nonanal, and heptanal, were significantly elevated in the breath of children infected with SARS-CoV-2, alongside other volatiles such as decane, tridecane, and 2-pentyl furan [9]. Notably, the overlap of octanal and heptanal in both adult and pediatric breath signatures suggests a shared metabolic response to viral infection, potentially linked to oxidative stress and lipid peroxidation pathways.

During the COVID-19 pandemic, trained detection dogs were deployed in numerous countries as a complementary diagnostic tool [11]. Initial deployments focused on testing biological samples collected in clinical settings, but later expanded to real-time screening at airports, public transport hubs, and large cultural events [11,12,13,14]. Multiple peer-reviewed studies confirmed that dogs could reliably differentiate between samples from SARS-CoV-2-infected and uninfected individuals. A systematic review of 27 studies from 15 countries found that, in the most rigorously designed trials, the sensitivity of canine olfactory detection ranged from 0.81 to 0.97, and the specificity from 0.91 to 1.0, frequently exceeding the performance thresholds set for rapid antigen tests [15]. These findings underscore the potential of trained dogs as a rapid, non-invasive screening tool, especially in high-throughput or resource-limited environments.

It should be emphasized that detection using trained scent dogs is significantly faster, more cost-effective, and environmentally friendly than standard laboratory-based diagnostics. For example, in a large-scale study conducted at four public concerts involving 2802 participants, dogs screened 40 sweat samples in under 60 s, enabling rapid prescreening with a high level of accuracy [11]. Similarly, in real-life airport screening at Helsinki-Vantaa International Airport, dogs achieved a diagnostic accuracy of 0.92 in validation trials and 0.977 agreement with RT-PCR in field deployment [14]. Remarkably, there are documented instances where dogs signaled samples as positive despite a negative RT-PCR result—only for the individual to test positive by PCR two to three days later [11]. This suggests that dogs may detect early-stage changes in VOC profiles—possibly during the presymptomatic or eclipse phase of infection—before viral RNA reaches levels detectable by standard tests. Such early detection capabilities underscore the potential of canine olfactory screening in preclinical identification of infection, especially where rapid, large-scale testing is required. While the per-test cost can be relatively low, the training, certification, and ongoing maintenance of detection dogs (handlers, housing, veterinary care) can be substantial and should be factored into implementation plans.

Although trained dogs have demonstrated high sensitivity and specificity in detecting SARS-CoV-2 infections, the precise olfactory cues they rely on remain poorly understood. It is not yet clear whether their detection ability is driven by the presence of distinct marker compounds generated by infection-induced metabolic changes, or rather by shifts in the relative concentrations of volatile compounds already present in healthy individuals. To date, studies have typically employed either canine olfactory detection or analytical chemistry approaches (e.g., GC-MS, IMS) in isolation. However, there is a lack of integrative research directly linking the dogs’ behavioral responses with the chemical profiles of the samples they assess. Addressing this gap is critical to elucidating the mechanisms underlying canine detection and to potentially identifying diagnostically relevant VOC signatures.

Recent studies have primarily focused on coronavirus samples during the pandemic. However, SARS-CoV-2 is classified as a BSL-3 pathogen, requiring stringent sample handling protocols and specialized laboratory facilities. Moreover, SARS-CoV-2 has been demonstrated to infect dogs, which could prove problematic in the experimental setup [16]. Further, all samples subjected to COVID-19 testing have to undergo thermal or chemical inactivation that could hamper the results [17]. In contrast, murine hepatitis virus strain 1 (MHV-1) is a strain of species *Murine coronavirus*, a BSL-2 pathogen that exclusively infects mice and is pathogenic only in certain laboratory mouse strains. As a member of the family *Coronaviridae* and the genus *Betacoronavirus*, similar to SARS-CoV-2, MHV-1 represents a suitable model for studying coronavirus infections [18,19].

**Figure 1:**
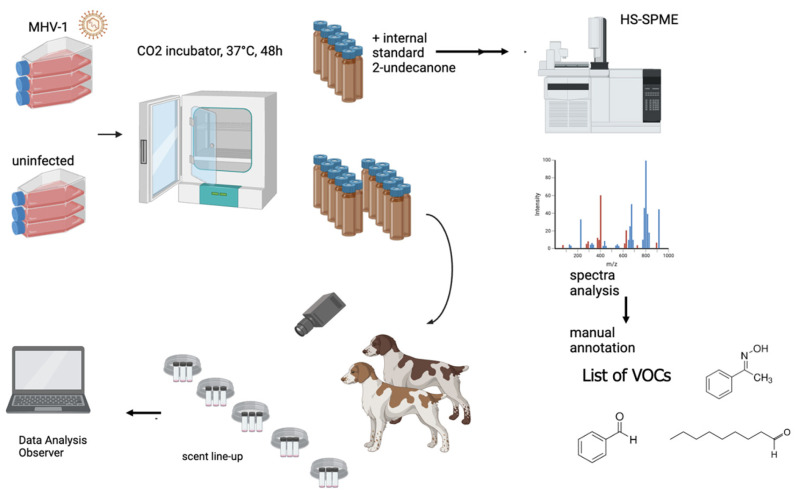
The workflow of the study. Cell cultures infected with murine hepatitis virus strain 1 (MHV-1) and uninfected controls were used as odor sources in double-blind discrimination tasks with trained detection dogs. In parallel, volatile compounds were analyzed by SPME–GC-MS. This combined approach enabled correlation of canine responses with VOC profiles to identify infection-related scent signatures.

The aim of this study was to identify the VOCs emitted by murine cell cultures infected with murine hepatitis virus strain 1 (MHV-1), in comparison to uninfected controls, and to evaluate whether trained detection dogs can discriminate between these two sample types based on differences in VOC profiles. Specifically, the study sought to test the hypothesis that canine detection relies not on the presence of unique marker compounds, but rather on changes in the concentrations and ratios of commonly occurring VOCs. By integrating chemical analysis of headspace volatiles with canine olfactory testing, the study provides a novel approach to exploring the mechanistic basis of scent-based detection. The workflow of this study is depicted in Figure 1.

## 2. Materials and Methods

### 2.1. Sample Preparation

Murine hepatitis virus strain 1 (MHV-1) (Bei resources) was titrated via the TCID50 method. Fifteen T175 flasks of 17CL-1 cells (a continuous mouse fibroblast cell line) were cultured and either infected with MHV-1 at a multiplicity of infection of 0.1 or left uninfected in control medium consisting of DMEM supplemented with 0.5% fetal bovine serum (FBS), 1% penicillin–streptomycin, and 1% L-glutamine (25 mL of medium in one flask). All flasks were set up on the same day and incubated in the same humidified 37 °C incubator with 5% CO_2_. At 48 h post-infection, upon observation of mild cytopathic effect, both infected and control cultures were subjected to two freeze–thaw cycles to release intracellular contents. For each condition (infected and control), cell suspensions and supernatants from all flasks were pooled prior to aliquoting and then aliquoted into 200 µL portions in sterile glass vials (5 mL ND18, 40 × 20 mm, Labsolute, Th.Geyer, Renningen, Germany) for canine olfactory training or 1 mL portions in chromatography vials (10 mL, ND18, 46 × 22.5 mm, Labsolute, Germany) for analysis by gas chromatography. Across all flasks, we used the same cell passage, identical plasticware, the same lot of medium and all supplements (including FBS), and a single, titered virus stock for infection. A total of 1200 uninfected and 600 virus-infected samples were prepared for the olfaction experiments. All samples were stored at −80 °C until further use.

### 2.2. Dogs

The study involved two domestic dogs of the Polish Hunting Spaniel breed (Polski Spaniel Myśliwski), a male (dog 1, Yoda) and a female (dog 2, Sara), both intact (not neutered/spayed), purchased from the registered kennel “Dwór Hanna.” At the commencement of training, both dogs were 2 years old. The dogs were in good health, with no known sensory or behavioral impairments, and lived in home environments with their respective trainers, who also served as their primary handlers throughout the study. Each dog completed two separate detection tests. The first test was conducted with cinnamon odor, which served as a benchmark to confirm that the dogs had correctly learned the detection task. The second test was carried out with virus-infected samples, where dogs were required to discriminate between infected and uninfected cell culture samples. Thus, every dog participated in both tests, in this fixed order: first cinnamon, then virus.

### 2.3. Canine Training Protocol

Detection dogs were trained using a positive reinforcement protocol based on standard operational odor detection training. The training procedure consisted of two stages: training on cinnamon odor (stage I) and training on MHV-1-infected samples (stage II).

Stage I—Cinnamon Odor Training: In the initial phase of training dogs for olfactory detection of viral particles, a foundational scent discrimination protocol was employed using cinnamon as a proxy target odor. Training was conducted in domestic settings by dog trainers who were also the animals’ primary caretakers, facilitating continuity and individualization of training. The training procedures encompassed two primary learning processes. First, classical conditioning was employed by pairing the conditioned stimulus (the scent of cinnamon) with an unconditioned stimulus (such as the scent of food or a toy), thereby establishing an initial positive emotional and attentional response to the target odor. Second, operant conditioning was used to shape and reinforce a specific trained final response (TFR): sitting for 2 s next to the bowl with the target odor (cinnamon). The TFR was strengthened exclusively through the use of positive reinforcement, utilizing food rewards or play to increase the likelihood of accurate and consistent responses.

Upon voluntary demonstration of the TFR in the presence of the cinnamon sample, dogs received a high-value reward to enhance learning through differential reinforcement. This trained response was then generalized across multiple contexts, with systematic introduction of environmental distractions (an empty sample vial, two different types of dog treats, and clove buds) to promote behavioral resilience and maintain detection accuracy under varied conditions.

For this stage each trainer worked independently, progressing at their own pace in home-based settings tailored to their individual dog’s needs.

The training outcomes on the cinnamon were assessed in laboratory settings (a scent detection lineup composed of five bowls, 60 cm apart (Supplementary Figure S1)) and recorded. The source of cinnamon in laboratory settings was constant (stored at room temperature). Correct identification required the dog to reliably indicate the single positive sample among four distractors. Once the dogs consistently achieved a minimum of 0.95 true positive indications in this 1-in-5 lineup configuration, they proceeded to the second phase of training (on virus samples).

Stage II—Virus Scent Training: In this stage, the virus sample odor (MHV-1, open glass vial) was initially combined with the cinnamon odor (cinnamon stick in separate vial). Dogs were required to achieve at least 13 true positive indications over 15 trials, each comprising five samples (with one positive sample per trial). Upon reaching this criterion, subsequent trials presented only the virus odor. After maintaining a minimum of 13 true positive indications in a series of 15 trials (each with five samples, one positive), the final training phase commenced by introducing negative samples (uninfected cell samples) and positive samples with MHV-1 virus only (without cinnamon odor).

Virus Test Protocol: Detection tests consisted of five samples arranged in a scent lineup—one true positive (MHV-1-infected), one blank (empty vial), and three true negatives (uninfected). Each test comprised four sessions of five runs each, with each run lasting from a few seconds to 2 min. There was a 5 min break between trials and a 30 min break between sessions, during which the dog rested, played or walked with its handler.

In both tests—the cinnamon detection benchmark and the virus detection test—the positive samples were presented in positions 1 through 5 an equal number of times. This balanced randomization minimizes the effect of sample position on overall latency.

During training and tests, dogs received positive reinforcement upon correctly identifying the target sample. Incorrect indications or failure to respond triggered a no-reward signal, prompting the dog to leave the training room before proceeding to the next run. All tests were conducted under double-blind conditions, meaning that neither the dog, the handler nor the person evaluating the results knew the location of the target sample (Supplementary Figure S1). When the dog correctly indicated the target, a reward signal was provided by the test operator, who observed the lineup via camera. When the handler heard the reward signal the dog was provided with positive reinforcement.

Our olfaction dog training program adhered to high ethical standards (decision of the local ethics committee: 071/2023).

### 2.4. Behavior Analysis

To systematically document and analyze the dogs’ behavioral responses during testing, all sessions were video-recorded and subsequently annotated manually, with latencies measured to the nearest second.

**Figure 2:**
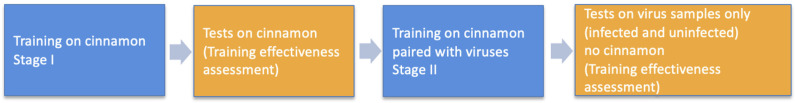
The workflow of the training and test design.

The latency was defined as the time (rounded to the nearest second) elapsed between the start of a test and the correct indication of the target sample. Trials in which the dog failed to identify the correct target odor were recorded as having a latency of zero and excluded from latency comparisons. Dogs were allowed to revisit samples within a lineup before giving their TFR. A trial was considered complete only after the dog provided the TFR. When a dog revisited a sample that was subsequently indicated as the true positive, this behavior was recorded as a “return.” If the indication was incorrect, even during a return, it was classified as a “non-detect”. This approach was taken into account in the statistical analysis, ensuring that each TFR represented an independent observation. In this manuscript we only aimed to report and compare the results from two tests: the cinnamon detection test and the virus detection test (orange background in Figure 2). We did not present or analyze data from the training phase. Accordingly, the only behavior evaluated in this study was the clear “sit” as a signal indicating a sample—whether correct or incorrect.

### 2.5. Chromatography Analysis

#### 2.5.1. Headspace Solid-Phase Microextraction (HS-SPME)

Two 10 mL chromatography vials per infected and uninfected sample, 1 mL each, were subjected to chromatography analysis (two replicates per sample). Prior to analysis, 10 µg of 2-undecanone (≥99% purity; Sigma-Aldrich, Steinheim, Germany) was added as an internal standard to the frozen matrix. Volatile compounds were extracted using an SPME Arrow fiber (1.10 mm diameter, 20 mm length) coated with a DVB/CWR/PDMS phase (CTC Analytics AG, Zwingen, Switzerland). The fiber was conditioned at 250 °C for 2 min before each extraction and reconditioned for 3 min following desorption. All incubations and extractions were performed using a PAL autosampler (CTC Analytics AG, Zwingen, Switzerland) equipped with temperature-controlled modules. Samples were incubated at 70 °C for 15 min, followed by extraction at the same temperature for 30 min. Desorption was carried out in split 1 mode at 250 °C for 3 min. Helium of analytical-grade purity (99.99997%; Air Products, Warsaw, Poland) was used as the carrier gas at a flow rate of 1 mL/min. Each sample was analyzed in two technical replicates. Presented data are averages from 4 analyses.

#### 2.5.2. Detection and Separation of VOCs

VOCs adsorbed on the SPME fiber were analyzed using a Shimadzu GCMS-QP2020 system (Shimadzu Corporation, Kyoto, Japan) equipped with a single quadrupole mass spectrometer. Separation was achieved using a Zebron ZB-5 capillary column (30 m × 0.25 mm i.d. × 0.25 µm film thickness; Phenomenex, Torrance, CA, USA). The oven temperature was initially held at 50 °C for 3 min, then ramped to 150 °C at a rate of 5 °C/min, followed by an increase to a final temperature of 250 °C at 15 °C/min, which was maintained for 5 min. The total chromatographic run time was 34.67 min. The mass spectrometer operated in electron ionization mode at 70 eV, with the interface and ion source temperatures set to 250 °C. Data acquisition was performed in full scan mode over a mass range of *m*/*z* 40–400, with spectra collected between 1 and 30 min of the chromatographic run.

#### 2.5.3. Determination of VOCs

Identification of VOCs was carried out by comparing the experimentally obtained mass spectra and calculated linear retention indices (LRIs) with reference data. The LRIs were determined using a standard mixture of n-alkanes (C7–C30; Sigma-Aldrich, Steinheim, Germany) run under identical chromatographic conditions. Mass spectral data were interpreted using automated and manual matching against multiple spectral libraries, including the NIST WebBook, NIST23 databases. Where applicable, additional verification was performed through comparison with published literature data. Compounds were considered to be identified when both the mass spectrum match and LRI deviation fell within accepted confidence thresholds. According to established guidelines and recommendations from research groups specializing in gas chromatography, the difference between experimental and literature retention indices should not exceed 10 units [20]. Therefore, we adopted a threshold value of 10 for this study. This integrative approach enabled reliable annotation of VOCs present in the headspace of both virus-infected and uninfected cell cultures. Before analysis, samples were checked in the absence of 2-undecanone, which was used as an internal standard.

### 2.6. Statistical Analysis

Revisit frequency and false positive rates were compared between virus and cinnamon conditions using independent-sample *t*-tests. For latency measures, analyses were performed both across and within individual dogs with *t*-test. The difference in false positive rates between virus and cinnamon groups was evaluated using a chi-square test. Statistical significance was set at *p* < 0.05. Those statistical analyses were conducted in Python version 3.13. Data preprocessing was performed using the pandas library, while inferential statistics were implemented with SciPy version 1.16. Data visualization and figure generation were carried out using Matplotlib version 3.10 and Seaborn version 0.13.

To evaluate whether response latency changed systematically across the testing sequence, a nonparametric correlation analysis was performed between trial number and latency for each dog and odor condition. Spearman’s rank correlation test was applied using GraphPad Prism version 10.4.1. This approach allowed assessment of potential monotonic trends in latency over time, independent of data normality. Separate analyses were conducted for each dog within both the cinnamon and virus testing groups. Statistical significance was defined as *p* < 0.05.

Statistical analysis of VOC data was performed using tailor-made Python scripts. The same as for revisit statistics, data handling utilized the pandas library, and statistical testing employed SciPy. Among several algorithms tested, a Random Forest classifier was selected for its robustness to noisy and unbalanced data, as well as the interpretability of its feature importance parameters.

## 3. Results

### 3.1. Dog Olfaction Training and Tests

During Phase II of the training, which involved discrimination between MHV-1-infected and uninfected cell culture samples, we observed that the dogs were unable to reliably differentiate the scent when presented with single vials containing 200 µL of cell culture material. Consistent olfactory discrimination emerged only when the three vials were placed in the detection bowl, suggesting that a minimum sample volume of approximately 600 µL (corresponding to ~0.6 g of material) was required to produce a detectable and distinct olfactory signal.

To evaluate diagnostic performance, sensitivity and specificity were calculated for each dog and condition. Sensitivity, defined as the proportion of correctly identified positive samples out of all positive presentations, was higher in the virus group (0.95) than in the cinnamon group (0.88). Dog 1 achieved a sensitivity of 1.00 in the virus group and 0.85 in the cinnamon group, while Dog 2 maintained a consistent sensitivity of 0.90 across both conditions.

The difference in false positive rates between virus and cinnamon groups revealed a statistically significant difference between the groups (χ^2^ = 2.082, *p*-value 0.041, df = 74). Similarly, missed detections, defined as trials in which the dog failed to identify the positive sample before leaving the testing area, were quantified and compared between groups. In terms of detection sensitivity, defined as the proportion of correctly identified positive samples out of all positive presentations, the cinnamon group yielded an overall sensitivity of 0.88, with 36 true positives out of 41 trials. The virus group (MHV-1 scent) showed a higher sensitivity of 0.95, with 38 true positives out of 40 trials. Individually, Dog 1 demonstrated a sensitivity of 0.85 for the cinnamon group and 1.00 for the virus group, while Dog 2 maintained a consistent sensitivity of 0.90 across both groups.

**Figure 3:**
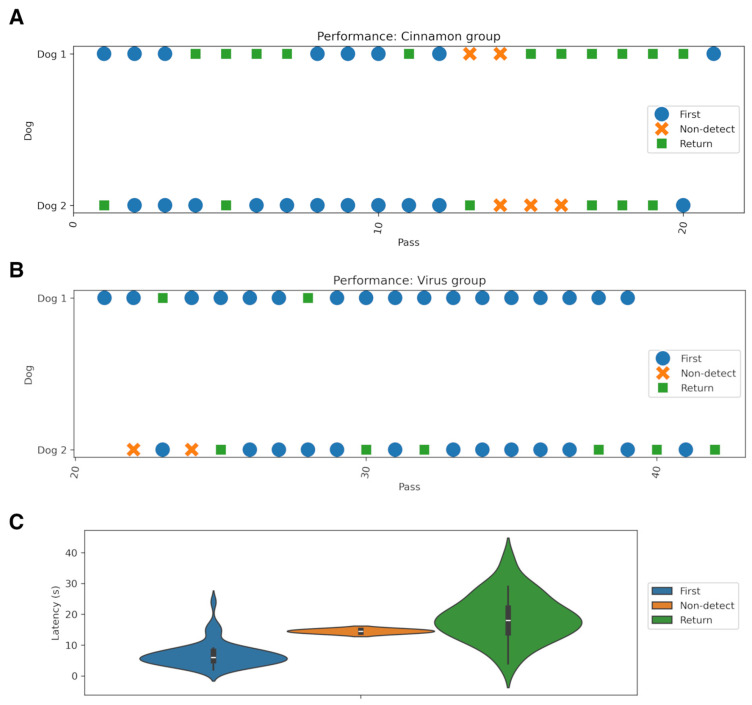
Detection performance across experimental conditions and detection latency for different prediction outcomes. In panels (**A**,**B**), the x-axis represents the sequential pass number, indicating progression through the experiment. Each point denotes a single trial, with marker color indicating detection outcome: correct detection on the first pass (blue), correct detection after returning to the sample (green), or failure to detect (orange). Panel (**C**) depicts detection latency for different prediction outcomes (violin plots). Latency for detection at a first attempt (blue, First), correct after return (green, Return). Latency of mistaken detection (orange, Non-detect) lies in between; however, due to the small number of mistaken detections, the result is not statistically significant (two-sided *t*-test).

In addition to detection outcomes, we examined the frequency of “revisit” behavior, operationally defined as the dog returning to re-examine the lineup after an initial pass (Figure 3). The occurrence of revisits differed between conditions, with a higher revisit rate observed in the cinnamon group. This difference was statistically significant (*t*-test statistic = 2.05, *p* = 0.044, df = 74).

The performance analysis presented in Figure 3A,B shows that in the virus test group, the majority of trials resulted in correct indications on the first attempt, whereas in the cinnamon group, a greater proportion of trials required a return to the target or resulted in missed detections.

**Figure 4:**
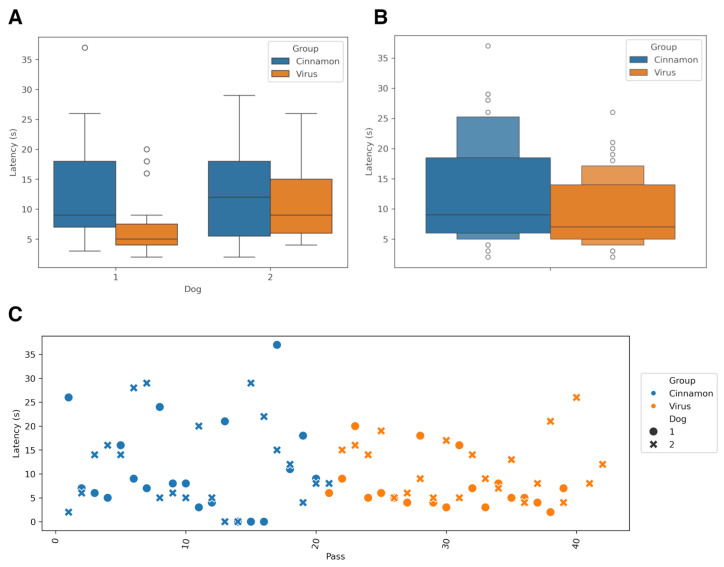
Distribution of detection latency across experimental conditions. Panel (**A**) illustrates the detection latency for individual trials, stratified by sample type (cinnamon test vs. virus-infected test) and dog identity. Circle: dog one, cross dog 2. Panel (**B**) summarizes the overall distribution. In panel (**C**), detection latency is plotted against the pass number within the test.

When considering both dogs together, mean detection latency was significantly shorter in the virus group compared to the cinnamon group (two-sided *t*-test, *p* = 0.04) (Figure 4B). When stratified by individual animal, Dog 1 exhibited a statistically significant reduction in detection latency in the virus test (*p* = 0.029), whereas Dog 2 showed no significant difference between test conditions (*p* = 0.46). Latency comparisons between dogs revealed no significant differences in the cinnamon group test (*p* = 0.95); however, in the virus group test, latency differed significantly between individuals (*p* = 0.03).

To assess whether response latency changed systematically over the course of testing, correlation analyses were conducted between trial number and latency for each dog and odor condition. In the cinnamon group, no significant relationship was observed (Dog 1: *r* = 0.2001, *p* = 0.4384; Dog 2: *r* = 0.0088, *p* = 0.9757). Similarly, in the virus group, no statistically significant correlations were detected (Dog 1: *r* = 0.3420, *p* = 0.1518; Dog 2: *r* = −0.1341, *p* = 0.5622). These results indicate that response latency did not systematically change with trial order in either test condition, suggesting consistent performance across the testing sequence (Figure 4C).

Collectively, these results indicate that trained dogs were capable of discriminating between virus-infected and non-infected samples with high sensitivity and that olfactory differentiation was associated with measurable behavioral differences in latency.

### 3.2. GC-MS Analysis

The GC-MS analysis of VOCs in MHV-1-infected and uninfected murine cell cultures revealed distinct chemical signatures associated with viral infection. Compound concentrations were quantified by normalizing peak areas to the internal standard, 2-undecanone, allowing for semi-quantitative comparisons across samples. In total, 14 VOCs were detected in infected samples and 12 in uninfected controls, with several compounds showing substantial differences in abundance or presence/absence patterns, as depicted in Table 1.

Among the most discriminatory compounds, 3-heptanone and 1-nonanol were exclusively present in infected samples (Table 1). Notably, 3-heptanone was detected at concentrations ranging from 769 to 982 ng/mL, and was completely absent from the control group. Decanal showed a similarly informative pattern: while it was detected in only one control sample (at 78 ng/mL), its levels in infected samples were on average twice as high, further supporting its classification as an infection-associated VOC. Nonanal also exhibited a comparable enrichment, with its relative abundance approximately 2-fold higher in infected samples.

In addition to aldehydes, the aromatic compounds acetophenone and benzaldehyde were significantly elevated in the infected group. The mean relative abundance of acetophenone was approximately 50% higher in infected samples than in controls.

The Random Forest model (n = 100 estimators, maximum number of features = 7), trained to predict infection status from partial VOC profiles, achieved a mean cross-validated accuracy of 0.82 (SD = 0.25). Variable importance scores from the model emphasized 3-heptanone, decanal, nonanal and acetophenone as key discriminatory features (see “Importance” column of Table 1). This segregation confirms the presence of consistent, infection-associated shifts in VOC profiles, driven by both dominant and trace-level compounds.

In summary, the mass spectrometry data reveal that MHV-1 infection induces robust and characteristic changes in the VOC profile of murine fibroblast cultures. These changes include both the appearance of infection-specific compounds (e.g., 3-heptanone) and significant increases in host-derived aldehydes and ketones likely associated with oxidative stress and inflammation. The integration of statistical analysis, compound ratios, and machine learning confirms the reproducibility and diagnostic potential of these infection-induced volatilomic signatures.

## 4. Discussion

This study showed that MHV-1 infection of a mouse fibroblast cell line was associated with measurable alterations in VOC profiles that were detectable by trained scent detection dogs. The combined use of canine behavioral assays and GC-MS-based VOC analysis—an approach that has been applied in previous studies—enabled a complementary assessment of infection-associated chemical changes and their olfactory relevance [17]. Together, these findings support the feasibility of integrating biological scent detection with analytical chemistry approaches in studies of virus-induced VOC alterations, without implying direct mechanistic causality.

Some of the compounds identified by us in virus-infected replicate samples are consistent with prior reports of infection-induced VOC profiles: for example, 3-heptanone and decanal were previously identified in IAV-infected cells [21] and nonanal has been repeatedly detected at elevated levels in the breath of SARS-CoV-2-infected individuals [9,10,22]. Similarly, nonanal, dodecane, and benzaldehyde were observed in cell cultures infected with RSV and IAV [8]. These VOCs are often associated with oxidative stress and lipid peroxidation—processes common in the cellular response to viral infection—and may act as secondary messengers in inflammation-related signaling pathways [23,24]. Although decanal has been previously reported as elevated in infected cells, it was also detected in one of the control samples in the present study. Given the limited number of control samples, this observation cannot be excluded and indicates that decanal may not be exclusively infection-specific. Its presence may reflect baseline cellular metabolism or environmental background. Therefore, decanal should be interpreted with caution and cannot be considered a standalone biomarker of infection based on the current dataset.

Interestingly, 3-heptanone, detected in both biological replicates of infected samples, is absent from uninfected controls. This compound belongs to a family of heptanones, structurally related to 2-heptanone and 4-heptanone, which have recently been identified in the urine of SARS-CoV-2 patients and convalescents [25]. Given the close proximity of their linear retention indices (LRIs), it is plausible that these findings are chemically convergent. The elevated presence of dodecane aligns with observations in experimentally infected humans with rhinovirus [26], although this compound was not found in in vitro rhinovirus-infected cultures—highlighting the relevance of the host cellular response in VOC production.

While acetophenone has not been firmly established as a coronavirus-specific marker, its elevation may reflect infection-driven metabolic shifts. Chuachaina et al. [22] hypothesize that acetophenone may be a downstream product of the upregulation of resistin-like molecule alpha, a protein responsible for modulation of inflammation, suggesting a role in host inflammatory or immune signaling rather than direct viral metabolism. However, in our system (coronavirus-infected murine fibroblasts), the biochemical source of acetophenone remains unresolved as fibroblasts do not express resistin-like molecule alpha. Benzaldehyde, similarly elevated, has previously been implicated in lipid peroxidation pathways and has been reported in infected epithelial cultures and exhaled breath from patients with respiratory viral infections [27,28].

2-Ethylhexanol, which was observed at high abundance in the chromatographic analysis in this study, has been reported in several studies. For example, it has been detected in the headspace of SK-MES cells (a human lung squamous cell carcinoma cell line), where it was proposed as a potential biomarker for lung cancer [29]. It has also been identified in body fluids and various cell lines in the context of cancer [30]. However, this compound is also known to leach from plastic materials and may therefore represent an analytical artifact, for instance originating from cell culture flasks [31].

From a behavioral perspective, dogs trained on pooled replicates of MHV-1-infected samples achieved a sensitivity of 0.95, compared to 0.88 in the cinnamon test conditions, indicating effective scent-based discrimination. These findings are consistent with previous, although still limited, studies investigating canine detection of virus-infected cell cultures. The slightly better performance in the virus test may reflect an order effect, as the dogs gained additional experience and confidence in the detection task over time before progressing to the next training stage.

In one study, dogs trained to detect SARS-CoV-2-infected cell cultures achieved a mean sensitivity of 0.758 and specificity of 0.902 [32]. Similarly, in another study involving bovine viral diarrhea virus (family *Flaviviridae*), the first dog achieved a diagnostic sensitivity of 0.850, while the second dog reached 0.967. Both dogs demonstrated very high diagnostic specificity: 0.981 and 0.993, respectively [33].

The observation that mean detection latency was significantly shorter in the virus group compared to the cinnamon group (*p* = 0.04) (particularly for Dog 1) suggests that the volatile profile of virus-infected samples may have been more distinctive or salient to the trained dogs. Similar findings have been reported in recent studies showing that certain odorants are detected more rapidly or reliably than others, depending on their chemical composition, volatility, and perceptual salience [34,35]. Thus, the scent profile of infected cell cultures might have elicited faster behavioral responses due to its stronger olfactory cues.

Alternatively, the difference may partly reflect a training or order effect, as the dogs were more experienced and confident during the second testing phase. Increased task familiarity and reinforcement history can shorten response times even when odor difficulty remains constant. Therefore, the reduced latency in the virus group likely represents a combined influence of odor salience and progressive training effects, both of which can modulate canine detection dynamics. In addition, revisit behavior (“returns”)—when dogs briefly rechecked a sample before giving their final response—was observed more often during the cinnamon test, which supports the above conclusion.

Interestingly, the dogs required a pooled sample volume of at least 600 µL (three vials) to achieve consistent detection, suggesting that VOC concentrations in single-vial samples were near or below their olfactory detection threshold. Differences in latency between dogs in the test condition also highlight individual variation in olfactory performance. These observations raise intriguing questions about dogs’ subjective sensitivity or preferences for specific VOC profiles—an area worth further exploration in future ethological studies.

Broader literature on olfactory perception suggests that odor recognition in dogs and other mammals is not solely dependent on the presence of specific compounds, but also on their concentration and perceptual salience. While perceptual constancy enables recognition of many odors across a range of intensities, this does not hold true for all volatiles. For certain odorants, qualitative perception can change markedly with concentration, as has been demonstrated in both humans and animals [36,37]. It is therefore plausible that the VOC profile of MHV-1-infected cell cultures, when present at low concentrations in individual vials, was not only less detectable but may have been perceived as qualitatively different—or insufficiently distinct from the uninfected cell sample odor. Similar challenges have been documented in dogs detecting explosives, which may fail to indicate when presented with odor intensities outside their training range [38]. Our results suggest that the same principle may apply to biomedical scent detection: training and testing protocols should account not only for the presence or absence of diagnostic volatiles, but also for their intensity, which can critically influence detection accuracy and generalization.

This study’s innovation lies in its integrated approach. Previous studies on virus-infected samples have generally focused on either chromatographic analysis or canine scent detection, whereas our work combines both approaches. By combining both methods, we not only confirm that trained dogs can detect infection-induced olfactory cues, but also provide evidence of specific chemical compounds likely responsible for this discrimination. This integrative methodology may help bridge the gap between behavioral observations and biochemical mechanisms in olfactory diagnostics.

Nevertheless, several limitations must be acknowledged. The study employed only two dogs, limiting the generalizability of the behavioral findings. Furthermore, although gas chromatography coupled with SPME is considered a gold standard for VOC analysis, it is possible that certain compounds with low volatility or low abundance remained undetected. Finally, while MHV-1 serves as a safe and relevant model for studying coronavirus infection under BSL-2 conditions, extrapolation to human pathogens such as SARS-CoV-2 should be made with caution. Moreover, a limitation of this study is that each trial contained a positive sample, which reflects an operational detection scenario but does not allow direct assessment of false-positive responses in the absence of a target odor; future studies will incorporate target-absent trials to further strengthen discrimination testing.

Future work should incorporate larger sample sets and explore the impact of different cell lines and viral species to validate and extend these preliminary findings to assess if the dogs trained for olfactory detection may be responding to broader patterns of volatile abundance rather than isolated biomarker compounds.

## 5. Conclusions

In conclusion, our findings support the hypothesis that viral infection alters the VOC profile of host cells in a way that is both chemically identifiable and behaviorally detectable by trained dogs. The identified compounds—including medium-chain aldehydes and ketones—are consistent with those reported in other respiratory viral infections and are likely linked to oxidative metabolic processes. This study not only advances our understanding of the chemical signatures of infection but also highlights the utility of scent detection dogs as sensitive biosensors when paired with analytical chemistry.

## Figures and Tables

**Table 1:** Volatile organic compounds (VOCs) identified in uninfected and MHV-1-infected cell culture samples. The table presents mean concentrations (in ng/dL) of each compound in uninfected and virus infected samples. Ratio indicates abundance ratio (infected/uninfected). Only the compounds inferred to be significant (with signal to noise ratio higher than 9, above quantification limit) have been included in the table. A ratio value greater than 1 indicates increased abundance in infected samples. The final column shows the relative importance of each compound for infection status classification, as determined by a Random Forest model trained on VOC profiles.

No	Compound	Uninfected Concentration (Vial 1)	Uninfected Concentration (Vial 2)	Infected Concentration (Vial 1)	Infected Concentration (Vial 2)	Ratio	Importance
1	Decanal	78.1	0.0	131.5	120.0	3.220	0.176
2	Acetophenone	4518.1	4237.1	6751.8	6319.8	1.493	0.141
3	Nonanal	392.1	377.4	559.9	824.1	1.799	0.141
4	2-Decanone	2085.3	2606.2	2700.7	2758.8	1.164	0.129
5	3-Heptanone	0.0	0.0	768.5	982.4	-	0.106
6	Benzaldehyde	10,940.1	12,805.7	17,632.3	19,719.1	1.573	0.071
7	3-Octanol	1009.4	956.4	895.6	1003.8	0.966	0.059
8	Tetradecane	3238.2	3377.9	3440.7	3056.9	0.982	0.047
9	1-Octanol	1563.3	784.5	1099.5	1186.5	0.974	0.035
10	1-Dodecanol	793.2	960.2	880.9	919.7	1.027	0.024
11	1-Nonanol	0.0	0.0	0.0	269.8	-	0.024
12	3-Heptanol	3395.3	2182.4	2212.9	2721.5	0.885	0.024
13	Dodecane	3541.4	3041.2	3088.2	2700.6	0.879	0.024
14	2-Ethylhexanol	241,163.1	225,550.5	218,903.7	235,366.6	0.973	excluded
15	2-UD (IS)	100,000	100,000	100,000	100,000	1	0

## Data Availability

The chromatography dataset is available at https://doi.org/10.57755/f4tq-yk95. Data obtained during the dog test is available upon reasonable request.

## Supplementary Materials

The following supporting information can be downloaded at: https://www.mdpi.com/article/10.3390/ani16040647/s1, Figure S1: (A) Picture of the 5 bowl line set up. (B) The double blind scenario, the researcher is sitting in separate room. (C) Dog one target odor marking. (D) Dog 2 target odor marking. (E) The design of the bowl with openable bottom part and holes in the upper part. (F) Table of training and expertimental setting.

## Abbreviations

The following abbreviations are used in this manuscript:

MHV-1	Murine hepatitis virus type 1
GC-MS	Gas chromatography–mass spectrometry
BSL-2	Biosafety level 2
BSL-3	Biosafety level 3
AUROC	Area under receiver-operator curve
RSV	Respiratory syncytial virus
IAV	Influenza A virus
VOC	Volatile organic compound
SARS-CoV-2	Severe acute respiratory syndrome coronavirus 2
IMS	Ion mobility spectrometry
SPME	Solid-phase microextraction
