# Supplementary material for: Trained Scent Dog Detection and GC-MS Analysis of Volatile Organic Compounds from Murine Coronavirus-Infected Cell Cultures
Source: Animals (Basel). 2026 Feb 18;16(4):647. doi: 10.3390/ani16040647 (PMC12937300; doi:10.3390/ani16040647)
Supplement: Supplementary file 1 [file animals-16-00647-s001.zip › animals-4011979-suplementary.pdf]

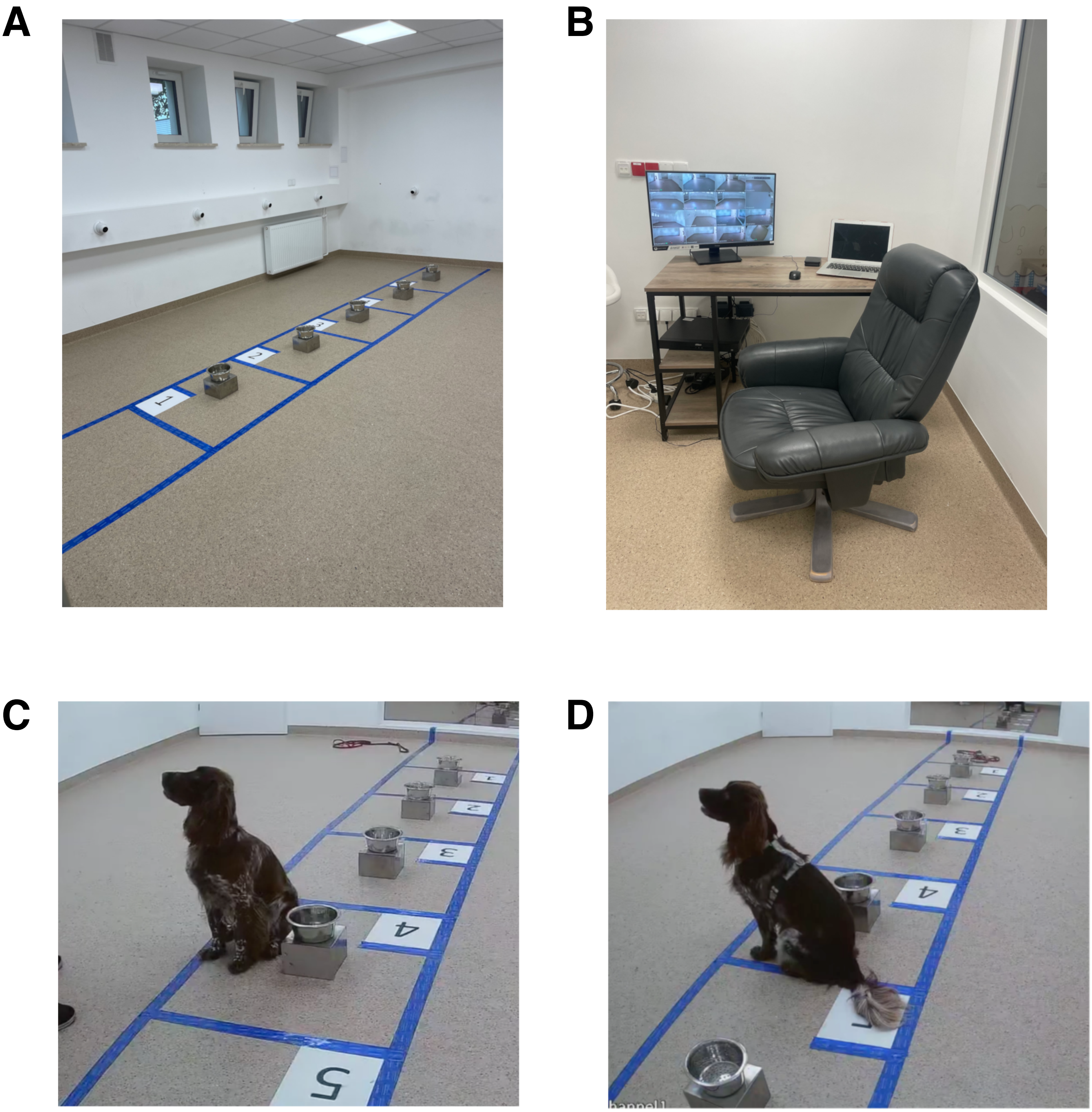

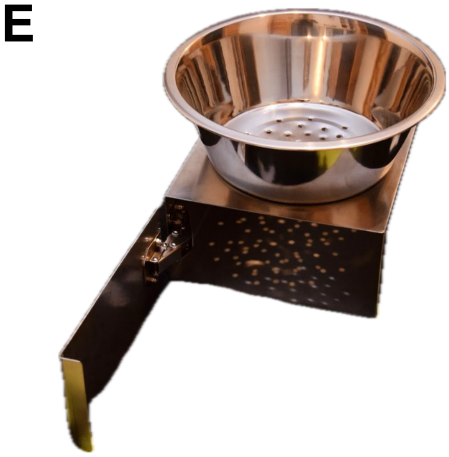

F

Phase	Day	Sample Configuration	Number of Trials	Rounds
Training	Day 1	1 × P+ (virus infected) + cinnamon, 4 × empty vial	20	4
Training	Day 2	1 × P+ (virus infected), 4 × empty vial	20	4
Training	Day 3	1 × P+ (virus infected), 1 × P− (non infected), remaining empty vial	20	4
Experimental	Day 4	1 × P+ (virus infected), 3 × P− (non infected), 1 × empty vial	20	4

Suplementary Figure S1
A Picture of the 5 bowl line set up. B The double blind scenario, the researcher is sitting in separate room. C Dog one target odor marking. D Dog 2 target odor marking .
E The design of the bowl with openable bottom part and holes in the upper part.
F Table of training and expertimental setting.
